# Upregulated expression of Annexin II is a prognostic marker for patients with gastric cancer

**DOI:** 10.1186/1477-7819-10-103

**Published:** 2012-06-08

**Authors:** Qi Zhang, Zaiyuan Ye, Qiong Yang, Xujun He, Huiju Wang, Zhongsheng Zhao

**Affiliations:** 1 Department of Surgery, Zhejiang Provincial People’s Hospital, Hangzhou, 310014, China; 2 Key Laboratory of Gastroenterology of Zhejiang Province, Hangzhou, 310014, China; 3 Department of Pathology, Zhejiang Provincial People’s Hospital, Hangzhou, 310014, China

**Keywords:** Annexin II, Prognostic, Gastric Cancer

## Abstract

**Background:**

The role of annexin II in the development and progression of gastric cancer was explored.

**Methods:**

Real-time PCR was conducted to detect annexin II and S100A6 mRNA expression. Protein expressions of annexin II and S100A6 were also examined by immunohistochemistry in 436 clinicopathologically characterized gastric cancer cases.

**Results:**

The expression of annexin II and S100A6 mRNA differ significantly among gastric tumor tissue and matched non-cancerous gastric mucosa. Protein levels of annexin II and S100A6 were up-regulated in gastric cancer compared with adjacent non-cancerous tissues. High expression of annexin II correlated with age, location of tumor, size of tumor, differentiation, histological type, depth of invasion, vessel invasion, lymph node metastasis, distant metastasis and Tumor, Node, Metastasis (TNM) stage, and also with expression of S100A6. Further multivariate analysis suggested that expression of annexin II and S100A6 were independent prognostic indicators for gastric cancer.

Cumulative five-year survival rates of patients with high expression of both annexin II and S100A6 was significantly lower than those with low expression of both.

**Conclusion:**

Expression of annexin II in gastric cancer was significantly associated with depth of invasion, lymph node metastasis and distant metastasis, TNM stage, high S100A6 expression, and poor prognosis. Annexin II and S100A6 proteins could be useful prognostic marker to predict tumor progression and prognosis in gastric cancer.

## Background

Gastric cancer (GC) is the fourth most common malignancy and is ranked as the second most common cause of cancer-related deaths, with approximately 870, 000 new cases occurring yearly [1]. The geographic distribution of incidence and mortality of GC varies remarkably worldwide, with more than one-third of all gastric cancer cases occurring in China [2,3]. Different etiologic factors, such as *Helicobacter pylori* infection, a diet high in salt, smoking, and environmental nitrates, have been associated with the development of GC [4]. Mortality from GC has declined for the past decades, mainly due to its early detection by endoscopic techniques, advances in chemotherapy and surgical techniques [5]. However, the overall five-year survival rate in China is only 40% [6]. More than 30% of surgical patients are too advanced to receive curative resection [7]. Therefore, identification of new diagnostic and prognostic biomarkers for cancer diagnosis and novel therapeutic targets for treatment are major goals in this field.

Annexins comprise a multigene family of soluble proteins which interact in a Ca-dependent manner with phospholipids and intracellular membranes [8]. Annexin II is an inducible, calcium-dependent phospholipid-binding protein which has overexpression in a variety of human malignancies and has emerged as an attractive candidate receptor for increased plasmin generation on the tumor cell surface [9-13]. It plays multiple roles in regulating cellular functions, including angiogenesis, proliferation, apoptosis, cell migration, invasion and adhesion [13-17]. Frequent partners for the S100 proteins are members of the annexin protein family [18]. At least ten different annexin-S100 complexes have been characterized, and it has been reported that proteins from the annexins family and the S100 protein family can form heterodimer complexes, such as the interaction between annexin II and S100A6 [19].

We performed the current study to examine expression of annexin II and S100A6 in surgical specimens of gastric carcinoma, to explore a possible correlation between annexin II and S100A6 expression and clinicopathological parameters, and to elucidate the clinical/prognostic value of annexin II and S100A6 in GC.

## Methods

### Patients and tissue samples

GC tissues were obtained from gastrectomy specimens of 436 patients with primary gastric cancer subjected to curative surgical resection (median age: 60.0 years; range: 30 to 91 years; 311 men, 125 women) from the Department of Surgery, Zhejiang Provincial People’s Hospital from January 1998 to January 2004. All tissue samples were formalin-fixed, paraffin-embedded, clinically and histopathologically diagnosed at the Departments of Gastrointestinal Surgery and Pathology. The follow-up deadline was December 2008 and all patients had follow-up records for more than five years. Survival time was calculated from date of surgery to follow-up deadline or date of death, which was caused mainly by carcinoma recurrence or metastasis. Based on the Lauren classification, 223 cases were intestinal and 213 cases were diffuse gastric cancer. There were 55, 163, and 218 cases from the cardia, body, and antrum, respectively. According to the 2002 World Health Organization (WHO) histological classification of gastric carcinoma, there were 326 tubular, 16 papillary, 29 mucinous, 65 signet-ring cell, and 13 highly differentiated, 128 well or moderately differentiated, 293 poorly differentiated, and two undifferentiated adenocarcinomas, respectively. Ninety cases were categorized as stage I, 104 were stage II, 173 were stage III, and 69 were stage IV. There were 61 cases with distant metastasis. A total of 92 non-cancerous gastric mucosa were obtained from gastrectomies of adjacent gastric cancer margins of >5 cm. Patients with advanced-stage disease underwent routine chemotherapy after surgery, but no radiation treatment was given to any patients included in our study. We followed up all patients by consulting their case documents or by telephone. All of the tissue specimens were obtained for the present study with patient informed consent and the use of the human specimens was approved by the Zhejiang Provincial People’s Hospital Institutional Review Board.

### Real-time quantitative PCR

The expressions of annexin II and S100A6 in 40 tumor tissue samples and matched non-cancerous gastric mucosa were confirmed by real-time PCR. Total RNA was extracted by Trizol and cDNA was reverse-transcribed by RevertAid TM reverse transcriptase. Real-time PCR was carried out using the ABI PRISM 7700 Sequence Detection System (Applied Biosystems, Hangzhou, Zhejiang, China), 95°C for ten minutes followed by 40 cycles at 95°C for 15 seconds and at 60°C for one minute. The primers for GAPDH (224 bp) were 5′- TGAAGGTCGGAGTCAACGG -3′ (sense) and 5′- CTGGAAGATGGTGATGGGATT -3′ (antisense). The primers for annexin II (213 bp) were 5′- GATGTGAAACACTTTGCTCCT -3′ (sense) and 5′- ATAGCGACACTTGGATAGGGG -3′ (antisense). The primers for S100A6 (176 bp) were 5′- GGCTGATGGAAGACTTGGACC -3′ (sense) and 5′- TTACCCACCACTGGATTTGACTC -3′ (antisense). The expression of GAPDH was used to normalize that of the target genes. Each assay was done in triplicate, the average was calculated, the expression level of annexin II was expressed as 2–ΔΔCt, where ΔCt = Ct(annexin II)–Ct(GAPDH), and the expression level of S100A6 was expressed as 2–ΔΔCt, where ΔCt = Ct(S100A6) – Ct(GAPDH).

### Tissue microarray

The tissue microarray (TMA), containing 528 cases (436 cancer samples and 92 non-cancer tissue samples), was constructed as described previously [20,21]. Briefly, formalin-fixed, paraffin-embedded archival tissue blocks with representative regions were selected according to their matching H & E-stained slides. Core tissue cylinders were collected from individual paraffin-embedded gastric tumors (donor blocks) and arranged in recipient paraffin blocks (tissue array blocks). It has previously been proven that staining results obtained from different intratumoral areas in various tumors correlate well, so a core was sampled in each case. From every archival block, one cylinder of 2.0 mm diameter was taken from cancer tissues of each patient from representative areas and transferred to paraffin recipient blocks using a trephine. An adequate case was defined as a tumor occupying >10% of the core area. Four-micrometer-thick sections were consecutively incised from the recipient block and transferred to polylysine-coated glass slides. H & E staining was performed on tissue microarray for confirmation of tumor tissue.

### Immunohistochemistry

Immunohistochemistry was performed for each antigen to study altered protein expression in 92 non-cancerous gastric tissue controls and 436 gastric cancer tissues [6]. Briefly, TMA slides were baked at 60°C for two hours followed by deparaffinization in xylene and rehydrated in graded alcohol.

The sections were submerged into ethylenediaminetetraacetic acid (EDTA) antigenic retrieval buffer and microwaved for antigenic retrieval, after which they were treated with 3% hydrogen peroxide in methanol to quench endogenous peroxidase activity, followed by incubation with 0.3% bovine serum albumin to reduce background non-specific staining. Sections were incubated with rabbit anti-annexin II (Epitomics Biotechnology Inc, Burlingame, California, USA), or mouse anti-S100A6 (Santa Cruz Biotechnology Inc, Delaware Avenue, California, USA), at 4°C overnight. Negative controls were performed by replacement of the primary antibody with non-reacting antibodies of the same species. After washing, tissue sections were treated with secondary antibody. Slides were stained with 3, 3-diaminobenzidine (DAB) and counterstained with hematoxylin, then dehydrated and mounted. The membrane with annexin II was stained as buffy, while S100A6 was stained as buffy in cytoplasm and nuclei. The degree of immunostaining was scored independently by two pathologists blinded to the clinical outcome of the patients, based on the proportion of positively stained tumor cells and intensity of staining [22,23]. For each antibody preparation studied, the staining index was calculated as the product of staining intensity score and the proportion of positive tumor cells. The location of immunoreactivity (cytoplasmic, nuclear, or combined) was noted and the proportion of positively staining tumor cells was as follows: 0 for <5% positive tumor cells; 1 for 6% to 25% positive tumor cells; 2 for 26% to 50% positive tumor cells; and 3 for >51% positive tumor cells. Staining intensity was graded according to the following criteria: 0 (no staining); 1 (weak staining = light yellow); 2 (moderate staining = yellow-brown); and 3 (strong staining = brown). We use this method of assessment to evaluate annexin II and S100A6 expression in human nontumor mucosa and malignant lesions by determining the staining index with scores of 0, 1, 2, 3, 4, 6, or 9. An optimal cut-off value was identified as: a staining index score of ≥4 was used to define tumors with high annexin II and S100A6 expression, and a staining index score of ≤3 was used to indicate low annexin II and S100A6 expression.

### Statistical analysis

Statistical analysis was performed using SPSS13.0 software. Measurement data were analyzed using the Student’s *t* test, while categorical data were studied using the chi-square test or Fisher exact test. The influence of prognostic factors on tumor-related survival was assessed by Kaplan-Meier estimates; the log-rank test was used to compute differences between curves. The multivariate Cox proportional hazard regression model was performed to assess prognostic values of protein expression. Correlation coefficients between protein expression and clinicopathological findings were analyzed using the Pearson correlation method. A value of *P* < 0.05 was considered statistically significant.

## Results

### mRNA expression of annexin II and S100A6 in gastric tumor tissue and non-tumor tissue

**Figure 1:**
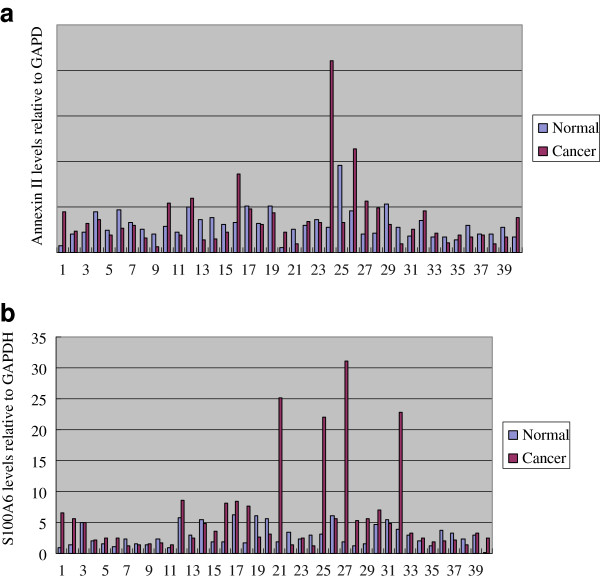
**Relative expression of annexin II and S100A6 in the corresponding gastric tumors compared with that in non-tumor tissues. a**. Relative expression of annexin II in the corresponding gastric tumors compared to non-tumor tissues. **b**. Relative expression of S100A6 in the corresponding gastric tumors compared to non-tumour tissues.

The mRNA expressions of annexin II and S100A6 in gastric tumor tissues and corresponding non-tumor tissues were analyzed using qRT-PCR in a total of 40 pairs of matched tissue specimens. In gastric tumors, the mRNA expression levels of Annexin II were higher in cancer tissues (0.037 ± 0.035) than non-tumor tissues (0.030 ± 0.016, *P* < 0.05, Figure 1A). The mRNA expression levels of S100A6 were higher (5.884 ± 6.988) than non-tumor tissues (2.883 ± 1.687, *P* < 0.05, Figure 1B).

### Expression of annexin II and S100A6 in GC and non-cancer mucosa

**Figure 2:**
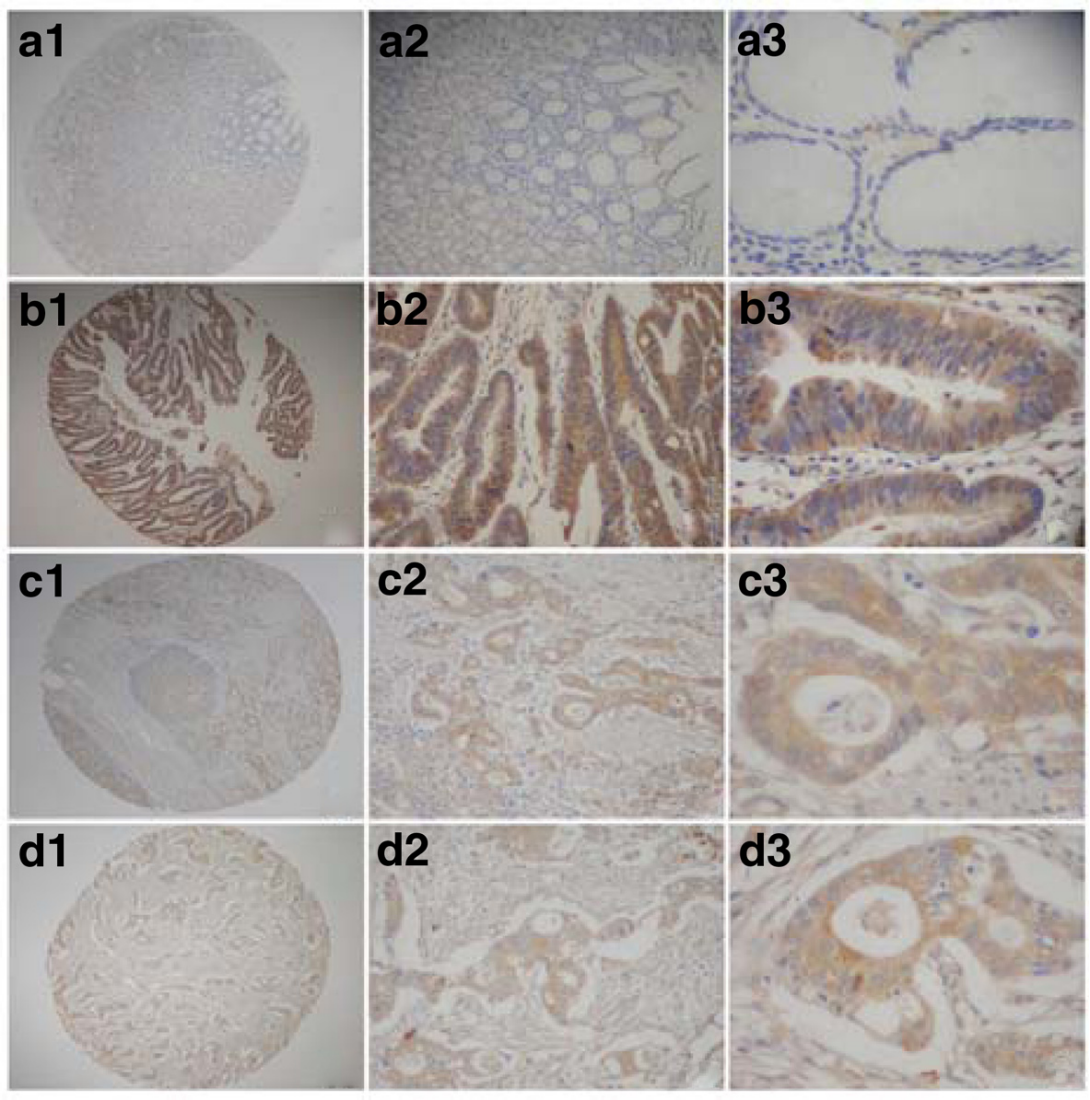
**Immunohistochemical staining for annexin II in gastric cancer lesions and noncancerous tissues.a,** 1 to 3, annexin II negative in noncancerous tissues; magnifications were × 40, ×100, and × 400, respectively. **b,** 1 to 3, annexin II was highly expressed in well differentiated adenocarcinoma; magnifications were × 40, ×100, and × 400, respectively. **c,** 1 to 3, annexin II was highly expressed in moderately differentiated adenocarcinoma; magnifications were × 40, ×100,and × 400, respectively. **d,** 1 to 3, annexin II was highly expressed in poorly differentiated adenocarcinoma; magnifications were × 40, ×100, and × 400, respectively.

**Figure 3:**
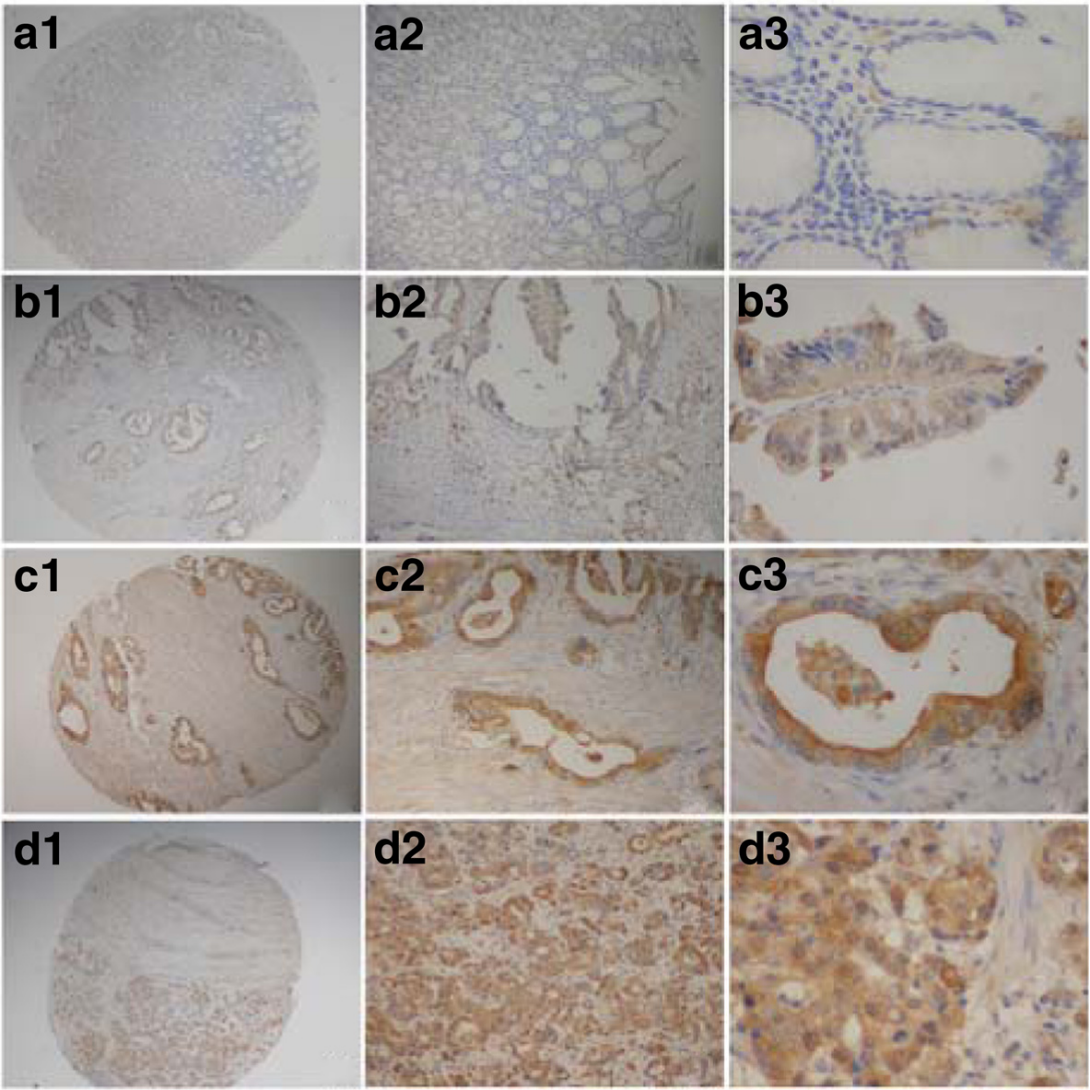
**Immunohistochemical staining for S100A6 in gastric cancer lesions and noncancerous tissues.a,** 1 to 3, S100A6 negative in noncancerous tissues; magnifications were × 40, ×100, and × 400, respectively. **b**, 1 to 3, S100A6 was highly expressed in well differentiated adenocarcinoma; magnifications were × 40, ×100 and × 400, respectively. **c**, 1 to 3, S100A6 was highly expressed in moderately differentiated adenocarcinoma; magnifications were × 40, ×100 and × 400, respectively. **d,** 1 to 3,S100A6 was highly expressed in poorly differentiated adenocarcinoma; magnifications were × 40, ×100, and × 400,respectively.

Annexin II protein was detected in 18 of 92 (19.57%) human non-tumor mucosa; all samples expressed the protein at low levels. Annexin II protein was detected in 175 of 436 (40.14%) human gastric cancer cases. High expression of annexin II protein was detected in 133 (30.50%) tumors, and low expression was detected in 42 (9.63%) tumors. Annexin II was overexpressed strongly in the cell membrane of primary cancer and weakly in the cytoplasm of carcinoma cells (Figure 2). S100A6 protein was detected in 9 of 92 (9.78%) human nontumor mucosa; all samples expressed the protein at low levels. S100A6 protein was detected in 256 of 436 (58.72%) human gastric cancer cases. High expression of S100A6 protein was detected in 183 (41.97%) tumors and low expression was detected in 73 (16.74%) tumors. S100A6 was predominantly localized in the cytoplasm, while nuclear staining was also detectable in some samples (Figure 3).

### Correlation between annexin II and S100A6 and clinicopathological features

The expression of annexin II correlated with age, location of tumor, size of tumor, differentiation, histological type, depth of invasion, vessel invasion, lymph node metastasis, distant metastasis, and TNM stage (*P* < 0.05) but did not correlate with gender (*P* > 0.05) (Table 1).

**Table 1:** Relationship of Annexin II expression with pathological parameters of tumor

**Clinical parameters**	**Annexin II**			
	**High**	**Low**	**t/χ**^**2**^	***P***
Age(years)	56.69 ± 11.94	62.57 ± 11.57	−5.098	0.000
Gender			0.812	0.367
Male	182(69.7%)	129(73.7%)		
Female	79(30.3%)	46(26.3%)		
Location			8.559	0.014
Proximal	23(8.8%)	32(18.3%)		
Middle	101(38.7%)	62(35.4%)		
Distal	137(52.5%)	81(46.3%)		
Size			32.555	0.000
<5cm	182(69.7%)	74(42.3%)		
≥5cm	79(30.3%)	101(57.7%)		
Lauren classification			10.277	0.001
Intestinal	142(54.4%)	122(69.7%)		
Diffuse	119(45.6%)	53(30.3%)		
Histology classification			7.658	0.054
Papillary adenocarcinoma	9(3.4%)	7(4.0%)		
Tubular adenocarcinoma	189(72.4%)	137(78.3%)		
Mucinous adenocarcinoma	13(5.0%)	16(9.1%)		
Signet-ring cell carcinoma	50(19.2%)	15(8.6%)		
Histologic differentiation			1.076	0.783
Well	12(4.6%)	1(0.6%)		
Moderately	69(26.4%)	59(33.7%)		
Poorly	179(68.6%)	114(65.1%)		
Others	1(0.4%)	1(0.6%)		
Invasion depth			61.527	0.000
T1	56(21.5%)	1(0.6%)		
T2	75(28.7%)	34(19.4%)		
T3	124(47.5%)	120(68.6%)		
T4	6(2.3%)	20(11.4%)		
TNM Stages			189.886	0.000
I	78(43.3%)	12(4.7%)		
II	71(39.4%)	33(12.9%)		
III	26(14.4%)	147(57.4%)		
IV	5(2.8%)	64(25.0%)		
Lymphatic metastasis			91.842	0.000
No	147(56.3%)	19(10.9%)		
Yes	114(43.7%)	156(89.1%)		
Regional lymph nodes			111.626	0.000
PN0	147(56.3%)	19(10.9%)		
PN1	71(27.2%)	65(37.1%)		
PN2	39(14.9%)	60(34.3%)		
PN3	4(1.5%)	31(17.7%)		
Distant metastasis^a^			30.214	0.000
No	244(93.5%)	131(74.9%)		
Yes	17(6.5%)	44(25.1%)		
S100A6			120.198	0.000
High	163(90.6%)	17(9.4%)		
Low	98(38.3%)	158(61.7%)		

^a^Distant metastasis refers to cancer that has spread from the original tumor to distant organs.

Positive expression of S100A6 correlated with age, location, size of tumor, depth of invasion, vessel invasion, lymph node metastasis, distant metastasis and TNM stage (*P* < 0.05), but did not correlate with gender, differentiation or histological type (*P* > 0.05) (Table 2). Factors with possible prognostic effects in gastric carcinoma were analyzed by Cox regression analysis. Statistical analysis showed that depth of invasion (*P* = 0.029), lymph node metastasis (*P* = 0.025), distant metastasis (*P* = 0.016), TNM stage (*P* = 0.021), expression of annexin II (*P* = 0.027) and expression of S100A6 (*P* = 0.011) were independent prognostic factors in patients with gastric carcinoma. However, the location of the tumor, tumor size, histological type, differentiation and vessel invasion had no prognostic value in our Cox’s proportional hazards regression model. It may relate to sample collection so it is critical that large sample and well-designed multicenter studies based on different groups are needed to confirm our results. We further performed the stratified analysis according to different TNM stages and the results confirmed that annexin II was an independent prognostic factor in patients with gastric carcinoma (*P* = 0.000).

**Table 2:** Relationship of S100A6 expression with pathological parameters of tumor

**Clinical parameters**	**S100A6**			
	**Negative**	**Positive**	**t/χ**^**2**^	***P***
Age(years)	57.17 ± 12.38	60.38 ± 11.78	−2.741	0.006
Gender			0.119	0.730
Male	130(41.8%)	181(58.2%)		
Female	50(40.0%)	75(60.0%)		
Location			6.130	0.047
Proximal	16(29.1%)	39(70.9%)		
Middle	63(38.7%)	100(61.3%)		
Distal	101(46.3%)	117(53.7%)		
Size			21.212	0.000
<5cm	129(50.4%)	127(49.6%)		
≥5cm	51(28.3%)	129(71.7%)		
Lauren classification			0.157	0.692
Intestinal	107(40.5%)	157(59.5%)		
Diffuse	73(42.4%)	99(57.6%)		
Histology classification			7.658	0.054
Papillary adenocarcinoma	9(56.3%)	7(43.7%)		
Tubular adenocarcinoma	134(41.1%)	192(58.9%)		
Mucinous adenocarcinoma	6(20.7%)	23(79.3%)		
Signet-ring cell carcinoma	31(47.7%)	34(52.3%)		
Histologic differentiation			1.076	0.783
Well	7(53.8%)	6(46.2%)		
Moderately	54(42.2%)	74(57.8%)		
Poorly	118(40.3%)	175(59.7%)		
Others	1(50.0%)	1(50.0%)		
Invasion depth			78.106	0.000
T1	51(89.5%)	6(10.5%)		
T2	62(56.9%)	47(43.1%)		
T3	65(26.6%)	179(73.4%)		
T4	2(7.7%)	24(92.3%)		
TNM Stages			185.557	0.000
I	78(86.7%)	12(13.3%)		
II	70(67.3%)	34(32.7%)		
III	27(15.6%)	146(84.4%)		
IV	5(7.2%)	64(92.8%)		
Lymphatic metastasis			110.475	0.000
No	121(72.9%)	45(27.1%)		
Yes	59(21.9%)	211(78.1%)		
Regional lymph nodes			121.478	0.000
PN0	121(72.9%)	45(27.1%)		
PN1	43(31.6%)	93(68.4%)		
PN2	13(13.1%)	86(86.9%)		
PN3	3(8.6%)	32(91.4%)		
Distant metastasis			28.936	0.000
No	174(46.4%)	201(53.6%)		
Yes	6(9.8%)	55(90.2%)		

### Association between expression of annexin II and S100A6

A total of 163 gastric cancer cases had low expression of both annexin II and S100A6, while 158 gastric cancer cases had high expression of both annexin II and S100A6. There was a significant correlation between annexin II and S100A6 (*χ*2 = 120.198; *P* = 0.000) (Table 1).

### Correlation between expression of annexin II and S100A6 and patient prognosis

**Figure 4:**
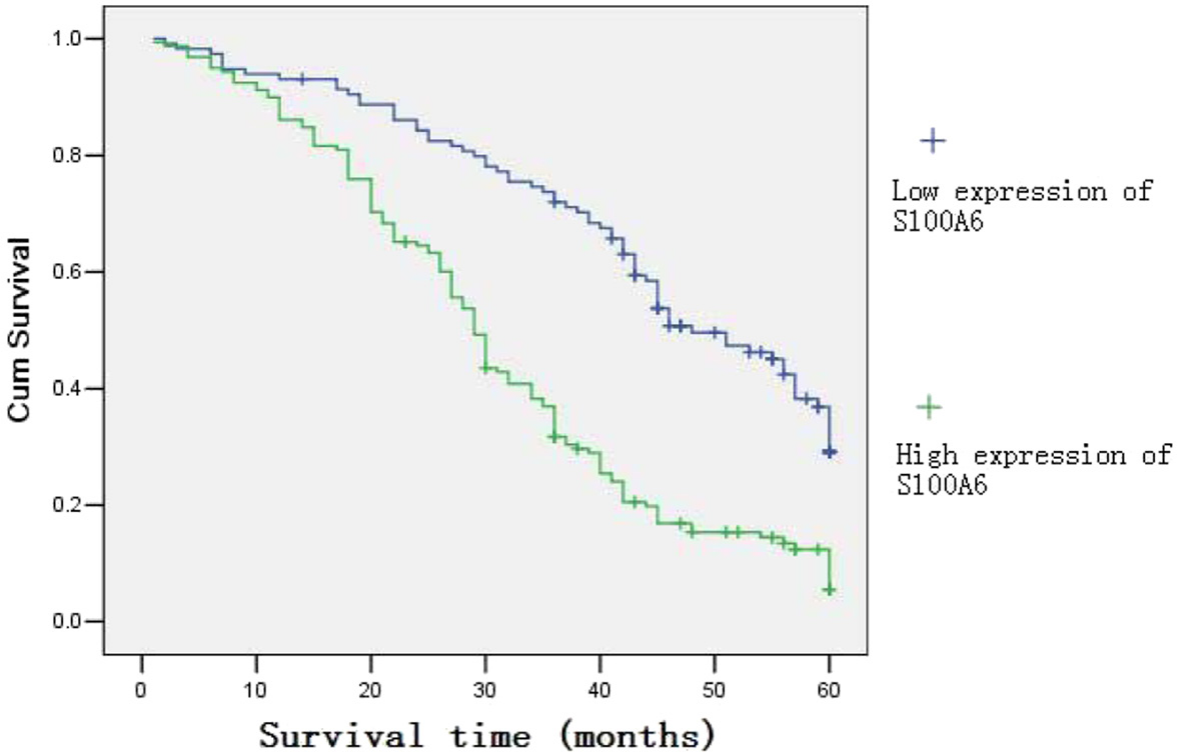
**Kaplan-Meier curves with univariate analyses (log-rank) for patients with simultaneously low annexin II and S100A6 expression versus high annexin II and S100A6 expression tumors in all gastric cancer.** The cumulative five-year survival rates of patients with a simultaneously high expression of annexin II and S100A6 were significantly lower than those in patients with simultaneously low expression (12.7% versus 38.3%, *P* = 0.000).

In stage I, II and III, the five-year survival rates of patients with high expression of annexin II were all significantly lower than in patients with low expression. In stage I, the cumulative five-year survival rate was 87.2% in the low-annexin II expression group, but 75.0% in the high expression group (*P* = 0.007). In stage II, the cumulative five-year survival rate in the low expression group (70.4%) was higher than in the high expression group (45.5%, *P* = 0.000). In stage III, the cumulative five-year survival rate was 30.8% in the low expression group, which was higher than in the high expression group (17.7%, *P* = 0.000). In stage IV, annexin II expression did not correlate with the five-year survival rate and the survival rate was 20.0% in the low expression group and 6.2% in the high expression group (*P* = 0.074). Cumulative five-year survival rates of patients with high expression of both annexin II and S100A6 was significantly lower than those with low expression of both (12.7% versus 38.3%, *P* = 0.000). (Figure 4)

## Discussion

Overexpression of the annexin II gene was observed in human gastric cancer and a recent report found that annexin II was significantly up-regulated in advanced gastric cancers and it could contribute to the progression of gastric carcinoma [24]. The possible clinical significance of annexin II has remained unclear in gastric cancer patients. Therefore, we used immunohistochemistry to examine the relationships between annexin II expression and the clinicopathologic characteristics of patients with gastric cancer.

The current study showed that annexin II is up-regulated in gastric cancer tissues compared to non-cancerous gastric mucosa. Annexin II levels were found to be correlated significantly with gastric cancer prognosis. In addition, high levels of annexin II protein expression in gastric cancer lesions were closely associated with age, location of tumor, size of tumor, differentiation, histological type, depth of invasion, vessel invasion, lymph node metastasis, distant metastasis, and TNM stage. Further multivariate analysis suggested that the depth of invasion, lymph node and distant metastasis, TNM stage, expression of annexin II, and expression of S100A6 were independent prognostic indicators for the disease.

Overexpression of annexin II has been associated with progression and metastasis in hepatocellular carcinoma, pancreatic cancer, colorectal cancer, lung cancer, breast cancer and renal cell carcinoma [9,11,25,26]. Conversely, down-regulation of annexin II has been reported in prostate carcinoma, esophageal carcinoma and laryngeal squamous cell carcinoma [27-29]. Therefore, the role of annexin II in cancer invasion and metastasis remains unclear. In hepatocellular carcinoma, annexin II is frequently up-regulated both on the mRNA and the protein level; hepatitis B virus (HBV)-induced hepatocellular carcinoma shows higher annexin II expression levels compared to hepatitis C virus (HCV)-induced hepatocellular carcinoma [9]. The up-regulation of annexin II expression in pancreatic, colorectal, and brain tumors was also directly correlated with advanced clinical stage [30]. Higher annexin II expression was observed in metastatic breast cancer and colon cancer cells compared with the non-metastatic cells [11,31]. A recent study using a proteomic approach investigating the secretome of the gastric cancer cell line SGC7901 identified annexin A2 as a secreted phosphoprotein [32]. Previous study has indicated that annexin II overexpression in gastric cancer was more frequently found in intestinal-type tumor cases, lymph node metastasis and venous invasion [24]. Together these studies suggest that annexin II has potential diagnostic and prognostic value as a therapeutic target to inhibit cancer progression and metastasis which needs to be further examined.

Accumulating evidence suggests that interactions between annexin II and its binding proteins play an important role in the tumor microenvironment and act together to enhance cancer metastasis [30]. The list of annexin II -interacting proteins includes t-PA, p11 protein, tenascin C and cathepsin B. Annexin and S100 proteins typically form complexes. Annexin II interacts with S100A4 as well as S100A10 [33]. It is highly expressed in endothelial cells and is overexpressed in breast and colon cancer, in which it has been implicated in invasive behavior. In both B-1 cells and stimulated B-2 cells, annexin II and S100A6 existed as Ca-dependent complexes [34]. It has also been reported that a strong association is between the presence of annexin II in the plasma membrane and high levels of cytoplasmic S100A6 of pancreatic cancer cells [35]. In MKN28 cells and primary gastric carcinomas, annexin II forms a complex with calpactin I light chain (P11) which belongs to the S-100 family as EF hand protein and is associated with cell differentiation, malignant transformation and cell cycle control [36-38].

To reveal the association among expression of annexin II and S100A6 in gastric cancer, we also studied the expression of S100A6 and, as we expected, S100A6 was up-regulated in gastric cancer tissues compared to normal gastric tissues. S100A6 protein levels were found to correlate significantly with the prognosis of gastric cancer. S100A6 was found to be widely expressed in gastric carcinomas and its expression could contribute to the progression of carcinomas and be useful in predicting the prognosis of gastric cancer.

The high level of S100A6 correlated with the expression of membranous annexin II in patient tumors while low S100A6 level correlated with a lack expression of membranous annexin II which suggested that S100A6 may affect the localization of annexin II to membranes [35]. The interactions between annexin II and S100A6 may play an important role in cancer metastasis and may provide a novel therapeutic approach for treating a variety of cancers. We also examined annexin II and S100A6 expression in gastric cancer specimens and its correlation with annexin II status. We found a positive correlation between annexin II and S100A6 expression with gastric cancer invasion and metastasis, suggesting that cells with positive annexin II expression may promote gastric cancer cell invasion and metastasis.

## Conclusions

In conclusion, our study identified up-regulation of annexin II and S100A6 in gastric cancer, including an assessment of annexin II and S100A6 expressions in gastric cancer tissues and noncancerous gastric tissues, suggesting that overexpression of annexin II and S100A6 are common features that may play an important role in the progression and metastasis of gastric cancer. The importance of annexin II and S100A6 up-regulation in gastric cancer is further highlighted by our results that correlate the unfavorable pathologic parameters of tumors with poor patient prognosis. These results suggest that annexin II and S100A6 may be useful as clinically significant prognostic and survival indicators. Our study has provided a basis for the development of novel biomarkers for the predicting progression and poor prognosis with gastric cancer.

## Abbreviations

bp, base pair; Ca-dependent, Calcium-dependent; cDNA, Complementary deoxyribonucleic acid; DAB, Diaminobenzidine; EDTA, Ethylene diamine tetraacetie acid; Epitomics Biotechnology Inc, Epitomics biotechnology incorporated; GAPDH, Glyceraldehyde-3-phosphate dehydrogenase; GC, Gastric cancer; H & E, Hematoxylin and eosin; HBV, Hepatitis B virus; HCV, Hepatitis C virus; mm, Millimeter; mRNA, Messenger RNA; PCR, Polymerase chain reaction; RNA, Ribonucleic acid; Santa Cruz Biotechnology Inc, Santa Cruz biotechnology incorporated; SPSS, Statistic package for social science; TNM, Tumor, Node, Metastases; TMA, Tissue microarray; WHO, World Health Organization.

## Competing interests

We declare that we have no conflict of interest.

## Authors’ Contributions

QZ, QY, XH, and HW participated in the scientific experiment. All the authors participated in the conception, the design, data collection and interpretation, manuscript preparation and literature search. All authors have read and approved the final manuscript.
